# Reviewing the availability and integration of community health information system for HIV/AIDS in Lesotho

**DOI:** 10.4102/jcmsa.v2i1.3

**Published:** 2024-05-09

**Authors:** Maseabata M. Ramathebane, Lineo J. Maja, Mosala Lets’olo, Sello Monts’i

**Affiliations:** 1 Department of Pharmacy, Faculty of Health Sciences, National University of Lesotho, Maseru, Lesotho; 2 Department of Economics, Faculty of Social Sciences, National University of Lesotho, Maseru, Lesotho; 3 Department of RISE COVID-19, Jhpiego, Maseru, Lesotho

**Keywords:** health information system, village health workers, community-oriented primary care, community clinical, non-clinical information

## Abstract

**Background:**

Universal Health Coverage (UHC) is an integral part of the Sustainable Development Goals, with community-based services playing a crucial role. Various stakeholders contribute to human immunodeficiency virus (HIV) interventions, which must be documented and shared with others for informed decision-making.

**Aim:**

This study aims to review the availability of a community health information system (CHIS) in Lesotho and its integration between the Ministry of Health (MOH) and the National AIDS Commission (NAC).

**Setting:**

The study is based on the Ministry of Health and the National AIDS Commission in Maseru, Lesotho.

**Method:**

A scoping review used peer-reviewed articles, documents from MOH and NAC, and other relevant reports from non-governmental organisations. The community information systems were examined for inclusion of clinical and non-clinical information. Possible linkages of information between MOH and NAC were reviewed.

**Results:**

Clinical information from CSOs is recorded in DHIS2 at the MOH facilities, while non-clinical information is reported in LOMSHA. However, clinical information from VHWs is currently not included in DHIS2 and formal reporting tools are being developed. There are no links between MOH and NAC, which limits information sharing.

**Conclusion:**

Although a CHIS exists, it lacks the necessary linkages. Community-based information from VHWs is not reported through DHIS2. Therefore, the country does not benefit fully from community-based health information.

**Contribution:**

Community health information systems review has never been conducted in Lesotho before. Therefore, this review will raise awareness about its importance and use in decision-making.

## Introduction

One of the core foundations of effective public health practice lies in providing precise and prompt data regarding health-intervention coverage, quality, and fairness. To accomplish this, community health data comprise one way to improve the standard of public health data overall.^1,2^ This is achieved by implementing a community health information system (CHIS), which effectively collects data in the field from community health workers (CHWs) and other providers who directly engage with the community.^1^ In the context of Lesotho, CHWs are referred to as village health workers (VHWs).

The CHIS is a health information system that connects various stakeholders and healthcare providers within a specific community.^3^ It serves as a locally driven and family-focused system for managing and overseeing the efforts of community workers in educating households and providing a comprehensive range of health services, including promotion, prevention, and basic curative care to families.^4^ Additionally, it has enhanced the decision-making process at the community level by offering decision-makers the necessary data and making the process more transparent.^5^

King et al. introduced a community health record framework, a multitiered, multisector model proposed to facilitate the development of a community health record.^6^ It describes an iterative and participatory process for achieving collaboration and information exchange between health care, public health and community organisations. One of the aims is to enable meaningful collaboration, which requires trust and time to develop and begins with focusing on a common purpose. The other aim is to facilitate a shared approach, which requires that stakeholders understand each of the problems and potential solutions from their perspective and the perspective of their collaborators. Furthermore, the framework aims to build workforce and infrastructure capacity by developing, implementing, and sustaining the community health record. This requires that multisector stakeholders develop the necessary epidemiology, informatics, and technical expertise and resources. Lastly, it aims to establish a new way of conducting business that enables the transformation of community health data into information and information into knowledge to aid decision-makers in collectively improving population health. Efficient and timely access to relevant multi-sector community health information depends on interoperability, the ability of different systems and organisations to exchange and use information easily.^6,7^

The national human immunodeficiency virus/acquired immunodeficiency syndrome (HIV/AIDS) strategic plan of Lesotho 2018 places community-level service delivery at the forefront of health service promotion, focusing on strengthening it to support the achievement of the 95-95-95 targets effectively.^8^ Various stakeholders, such as VHWs, community ART groups (CAGs), civil society organisations (CSOs), non-governmental organisations (NGOs), and community councils, play a role in delivering HIV interventions across the prevention, testing, and treatment continuum.^8^ To enhance the VHW programme, the Ministry of Health (MOH) has established a VHW policy and strategy.^9^ Additionally, key performance indicators have been instrumental in informing the development of data-collection tools for VHWs, and the VHW programme is currently working on creating a data-management system specifically for VHW information.^9^ These efforts align with the findings of the Jeremie et al. study, which emphasised the importance of an organised community information management system for health managers to plan and support community healthcare effectively, thereby benefiting primary healthcare.^10^

The availability of community data plays a vital role in facilitating the exchange of health information between the community and the healthcare system.^10^ Village health workers and health centre committees have led to increased coverage of services, making health services more accessible to the community. These services primarily focus on promotion, prevention and rehabilitation. Braun et al. highlighted the various functions CHWs performed, including conducting home visits, assessing and treating diseases, collecting data, providing education and counselling, and making referrals for further care.^11^ They also support TB and HIV patients by accompanying them to health facilities, ensuring timely medication pick-up, and promoting adherence. Furthermore, CHWs organise health education gatherings and facilitate immunisation efforts within their communities. This information must be compiled and shared with the MOH and other stakeholders to assess if targets have been met. Jeremie et al. emphasised the significant role of CHWs in gathering information at the community level.^10^ USAID states that a well-functioning health information system provides reliable and timely information on health determinants, health system performance, and overall health status.^12^ The production, analysis, dissemination, and utilisation of information form a crucial foundation for effective health systems.^12^

The National AIDS Commission (NAC) uses District Health Information System 2 (DHIS2), which is intended to collect non-clinical data from CSOs and community councils. This DHIS2 at NAC is called the Lesotho Output Monitoring System for HIV and AIDS (LOMSHA). Lesotho Output Monitoring System for HIV and AIDS was established to enhance the coordination of non-clinical data from all stakeholders providing HIV and TB services as per the Lesotho Essential Package of HIV Services (ESP).^13^ National AIDS Commission further articulates that organisations will be empowered with efficient accounting and reporting practices to deliver reliable and consistent information about HIV and TB.^13^ This will enable them to improve their capacity to provide accurate and up-to-date information on these diseases. However, there is no indication of information integration between DHIS2 and LOMSHA. Therefore, a lack of information sharing on community-based data from LOMSHA and DHIS2 may affect community-level interventions by MOH, and duplication of efforts may occur.

One notable strength is the training provided to programme managers, equipping them with the skills to generate various reports using the district health information system (DHIS2). Jeremie et al. further highlighted the training of CHWs to collect accurate data, compile it, and disseminate it is crucial for planning purposes and effective health interventions within the community.^10^ Evaluations have also been conducted on the use of mobile health (mHealth) among CHWs, addressing a range of infectious diseases such as tuberculosis (TB) and HIV. Ha et al. assessed the mHealth approach to TB contact tracing in Botswana, which resulted in a reduced median time for completing tracing and improved data collection.^14^ The usability and acceptability of the programme were also rated positively. Similarly, an intervention analysis in Nigeria (involving 203 participants) demonstrated modest improvements in health professionals’ behaviour (e.g. increased hand washing and disinfectant use) and knowledge of Ebola.^15^

In Kenya, Finocchario-Kessler et al. conducted an observational pilot study that integrated internet and SMS alerts with tracking, leading to improvements in care.^16^ These improvements included continued care for mothers, increased uptake of antiretrovirals for newborns testing positive for HIV, and faster delivery of test results. A systematic review by Ambia et al. identified 34 studies, indicating that mobile phone reminders could reduce mother-to-child transmission of HIV and improve early infant HIV diagnosis.^17^ A study conducted in Kenya involving 650 mothers demonstrated the potential of mHealth in reducing HIV transmission from mother to child.^18^ Another study in Sri Lanka explored mHealth use among 29 public health inspectors addressing dengue fever.^19^ They emphasised the need for collaborative and culturally specific programme development approaches.

In Lesotho, the VHW programme suffers from weak coordination and inadequate collaboration among partners, resulting in insufficient support for VHWs and fragmented programmes, activities, support and incentives. There is a shortage of policy guidelines and standardised reporting tools for community-based TB and HIV/AIDS care in districts. Thus, the aim of this study is to review the availability of a CHIS in Lesotho and its integration between the MOH and the NAC.

## Methods

### Study design

A scoping review was conducted focusing on CHIS in two study settings, which are Lesotho’s MOH and the NAC. Structurally, MOH and NAC are two different entities. The MOH oversees all health matters, including the clinical management of HIV and TB. The NAC is a coordinating body between CSOs and NGOs dealing with the non-clinical information management of HIV. National AIDS Commission is unique because it is under the Prime Minister’s office. There are two health information systems: the DHIS2 for MOH and the LOMSHA for NAC. The DHIS2 includes clinical information from MOH facilities, including primary health care (PHC) facilities. The LOMSHA captures non-clinical information from CSOs and community councils.

### Data sources and search strategy

For the scoping review, the search engines used were Pubmed or Medline, WHO Library, Science Direct, Google, Google Scholar and Cochrane or Wiley Library. The search terms were used to generate relevant articles, which were then reviewed for relevance using the inclusion criteria. Studies that met the inclusion criteria and answered the research questions were selected and used for this review. Search terms used included ‘community health information system LMIC’, ‘health information for community-based programmes’, ‘community-based information system in Lesotho’, ‘structure of community health information system’, ‘availability of community health information system’.

**FIGURE 1:**
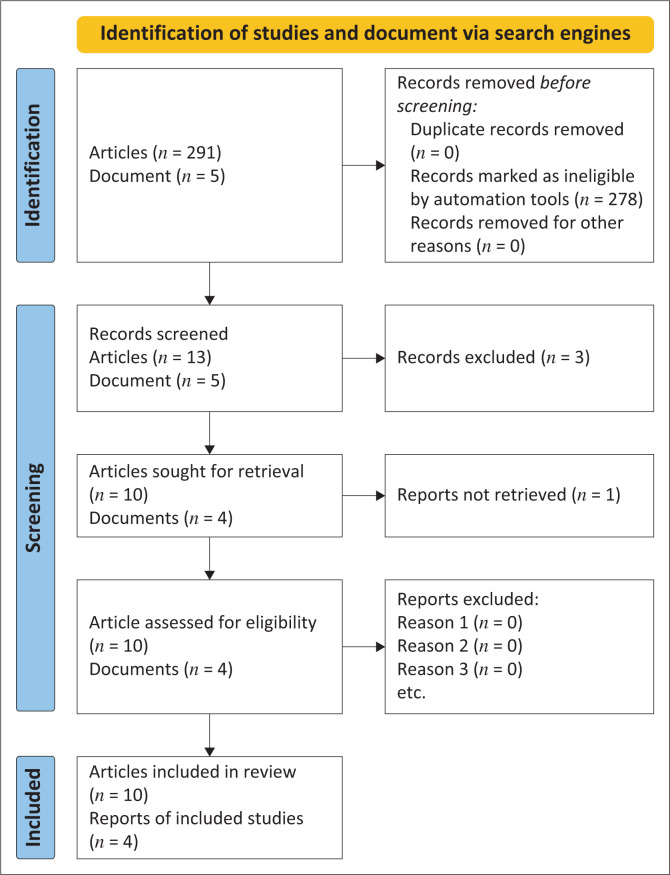
Schematic representation of study selection adopted from PRISMA adapted for this review.

HIV/AIDS Policy 2019, VHW Policy 2020, Lesotho HIV/AIDS Strategic Plan 2018–2023, and NAC 2017 served as additional data sources for this research (refer to Figure 1). The village health system network development in Lesotho was discussed with the VHW programme officer. The NAC officials supplied details about non-clinical CHIS.

The review questions include: Is a CHIS available in Lesotho? Is community-based information integrated between NAC and MOH Lesotho?

### Studies eligibility criteria

#### Inclusion criteria

- Lesotho MOH HIV or TB-related documents which included policies, strategic plans, health information system (HIS) documents, and periodic reports from 2015 to date. Village health workers-related documents, for example, policies.
- NAC HIV or TB-related documents which included policies, strategic plans, health information system (HIS) documents, and periodic reports from 2015 to date. CSOs-related documents, for example, policies.
- HIS and CHIS articles related to HIV and TB diseases in Lesotho from 2010 to date.
- WHO or UNAIDS reports that included Lesotho’s CHIS and HIS.
- Only studies written in English are available as full free texts.

#### Exclusion criteria

- MOH documents not related to HIV, TB, HIS, CHIS, or VHWs.
- NAC documents not related to HIV, TB, HIS, CHIS, community council, and CSOs.
- HIS articles related to other diseases, such as maternal health.

### Study selection and data extraction

Two review authors performed the initial searches. Screening of titles, abstracts and full texts was conducted by two review authors (M.L. and L.M.). A data extraction form was developed and used to summarise the study findings. Data extraction and critical appraisal were conducted for each included study or report. The data-extraction template included study characteristics: author or organisation, year, country, setting or population, study design, lesson learned and identified implementation challenges. The two authors (M.L. and L.M.) independently extracted data. The two authors discussed disagreements and consulted a third author (M.R.) for any persistent disputes.

### Analysis and reporting

The findings of this study were presented in two ways. Firstly, the study findings from the existing literature were shown in a table highlighting authors, year, study design, and issues on the availability of CHIS and information integration between NAC and MOH Lesotho refer to Table 1. Secondly, the narrative literature was structured around the themes derived from the study results. The themes included the current availability of CHIS in Lesotho, community clinical and non-clinical information, CHIS at the community level in Lesotho, and non-clinical health information systems at the community level in Lesotho.

**TABLE 1:** Characteristics of included studies for the review.

Author	Year	Country	Setting/Population	Study design	State of CHIS and Lessons Learned	Challenges identified
Mekonnen et al.^1^	2022	Ethiopia	21 articles	Scoping review	CHIS should enable information to be shared among community-based services and feed into national health management information systems The availability of information on health services performance can empower community health workers to improve the quality of community-based health services Technology plays a role in strengthening CHIS in most LMICs	This vital information on health services provided by CHWs is not routinely captured and integrated There are fragmented and disjointed efforts to strengthen community-based health information systems in LMICs A culture of information use needs to be reviewed.
Hampshire et al.^20^	2022	Ghana Malawi Ethiopia	80 CHWs 92 CHWs 63 CHWs	Mixed methods (qualitative and quantitative)	CHWs were using personal mobile phones to address gaps in healthcare infrastructure and resourcing. For example: facilitating regular patient communication, managing medicine supplies, compiling/reporting data, seeking advice on case management, liaising with volunteers, and responding to emergencies.	Costs and challenges associated with informal mHealth. CHWs reported difficulties in keeping phones operational where power outages and network unreliability were a problem. Phones brought financial burdens and the blurring of work/life boundaries. Security of personal data was also a concern for CHW. Weak regulation and enforcement can make digital health vulnerable to security breaches in some LMICs.
Birru et al.^21^	2023	Lesotho	**Evaluation participants** 12 facilities each in Butha Buthe and Mohale’sHoek districts 17 facilities in Berea district 25 facilities in Leribe district. **Key informant interviews (25):** 6 interviews each in Berea and Butha Buthe, 5 interviews each in Leribe and Mohale’s Hoek 3 interviews at the central MoH office	Mixed methods (qualitative and quantitative)	MOH had a strong data completion rate which was sustained despite an increase in service utilisation. The data quality improvement efforts were optimised through improved behavioural, technical and organisational factors. Vertical programmes such as HIV, TB and immunisation had a high data completion. Availability of reporting forms for community health workers to report indicators related to maternal and child health, HIV and TB programmes conducted in the community level. These reports were reviewed monthly by supervisors, health facility nurse in charge and public health nurse. HCWs highlighted improved organisational structure, staffing, training, data reporting and analysis processes at all levels. PHC review meetings are an essential part of facilitating improved data quality and data-utilisation practices.	DHMTs and facility-level staff faced major challenges coordinating donors who had different competing monthly report priorities.
Mwase et al.^22^	2010	Lesotho	52 health centres and filter clinics 21 hospitals 10 DHMTs	Mixed methods (qualitative and quantitative)	Weak and ineffective health information system Lesotho needs to develop standards for electronic systems to ensure a level of interoperability between systems.	Poor data use culture
Betjeman et al.^23^	2013	Sub-Saharan Africa	18 articles	Systematic review	mHealth programmes being used in concert with an EMR system could facilitate patient care coordination, health worker efficiency and data collection and analysis.	The lack of existing infrastructure in many areas of SSA, as well as the lack of background familiarity with EMR platforms among healthcare workers, poses significant obstacles to the adoption of EMR systems in many areas where mHealth may be currently viable.
Ngcobo et al.^24^	2022	Sub-Saharan Africa	17 articles	Systematic, aggregated, narrative review	mHealth improved the quality of services provided by CHWs. These mHealth interventions could improve reporting, communication, supervision, guide learning, and linkage to care.	Sustainability of CHW programmes is still a challenge, with many CHW programmes relying on donor support.
Källander et al.^25^	2013	LMICs	6 themes of mHealth initiatives	Thematic review	mHealth – CHWs used a combination of simple mobile phone applications for data submission, job aids to improve diagnostics, and sending and receiving SMS messages and reminders. Data feedback reports should be distributed to CHWs submitting data regularly (perhaps initially once per week)	Sustainability and scalability are still the main challenges to the strategic deployment of mHealth applications. Moving mHealth approaches from pilot projects to national scalable programmes while properly engaging health workers and communities.
Tsoeu^26^	2022	Lesotho	291 healthcare workers	Quantitative descriptive and cross-sectional design	Since the use of DHIS2 in Lesotho; there has been an improvement in the reporting rate and completeness of reports from facilities. DHIS2 is used for different reasons and these include data collection, reporting, data validation and decision-making.	In some health centres there are challenges of late reporting, inconsistency in data reports and DHIS2 reports, and data analysis function not effectively used. Lack of capacitated human resource has been the challenge hindering successful implementation of DHIS2 for many developing countries.
-	2016	Low- and middle-income countries	23 eligible references	Literature search	Community-Based HIS can be used for case management, to document individual needs to inform care plans, to enable bidirectional referrals, and to track patients lost to follow-up.	Community-Based HIS are faced with challenges such as lack of technical capacity of CWs (need for intensive training with periodic refresher courses for CHWs involved in data collection. Another challenge regarding the CWs or volunteers is the added workload of data collection and associated activities. Another challenge in adding to new CW responsibilities is the lack, or early stages, of integration of CBHIS in formal HMIS. Without complete integration, there are duplicative efforts in data collection, analysis and reporting.
-	2013	Africa Asia Latin America	Africa = 19 Asia = 5 Latin America = 3	Systematic review	mHealth technology was most commonly used for data collection, decision support, alerts and reminders, and information on demand, facilitating CHW activities associated with field-based research and direct provision of medical care. CHWs are using mHealth tools with increasing effectiveness to improve the delivery of maternal and child health, HIV and other sexual and reproductive health services, and other general health services in the developing world, mainly in Africa. Use of mobile technologies can be broadened to support and empower CHWs in their role as a bridge between formal health systems and communities. CHWs are increasingly using mHealth tools to enhance delivery of community-based healthcare services and to access continued learning.	Social, policy, and technical challenges remain. Greater than the challenges related to the technology design and computer engineering are the policy barriers to institutionalising CHWs as an accepted part of the health system.
-	2019	Lesotho	-	Report	The Lesotho Output Monitoring System for HIV and AIDS (LOMSHA) is in place at the community level as CHIS.	The DHIS2 now includes the HIV module. The weakness is that because of inadequate capacity in electronic record keeping country-wide, even with the use of unique identifiers, it is still a challenge to ensure that there is no duplication of some of the data.
National HIV and AIDS Strategic Plan^8^	2018–2023	Lesotho	-	Report	Community and other non-health sector routine data is processed and managed through the Lesotho Output Monitoring System for HIV and AIDS (LOMSHA), managed by the National AIDS Commission The LOMSHA is in place at the community level. DHIS2 will be updated to contain modules from LOMSHA.	DHIS2 and LOMSHA are not merged; leading to potential duplication and some inefficiencies in programme design and implementation. Enhance integrated reporting between community and government structures. CSOs at the community level will report through LOMSHA, which will, in turn channel information to the DHIS 2 Expand parameters or indicators for collection and integrate community-level information system (LOMSHA), including key population data, into DHIS. Standardise and digitise (computerise) the LOMSHA and HMIS systems and integrate them into the community at the tertiary hospital level. Build the capacity of the community health system (CSOs, FBOs, CBOs, CHW, PLHIV networks, KP networks etc.) in data collection
Lesotho HIV and AIDS M&E Plan^27^	2018-2023	Lesotho	-	Report	The National AIDS Commission, with the support of partners, has revamped the Lesotho Output Based System for HIV and AIDS (LOMSHA) to capture mainly data from the community or non-clinical interventions.	Communication between HMIS unit and the programmes needs further improvements through sharing of information, increasing inter-operability/linkages with different systems, the community information system (LOMSHA), introduction of modules for unique patient identification and capacity building of human resources through a concerted national effort shall be needed.
Village Health Programme Policy^9^	2020	Lesotho	-	Report	To ensure that VHP data is used coherently, cohesively, and effectively, the Village Health Programme collects data through a singular, harmonised data collection and reporting system in line with the national monitoring and evaluation (M&E) system. The VHP will work with the Directorate of Health Planning and Statistics to design reporting tools that are user-friendly and that will be aligned with various e-health systems and fully integrated with the national Health Management Information System (HMIS).	In terms of administration, management and operations, the health sector still has some limitations which negatively impact the quality of service delivery. These include weak and ineffective health information system and centralised decision-making.

CHW, community health workers; CHIS, community health information system; LMICs; low-or middle income countries; MOH, Ministry of Health; PHC, primary health care; EMR, electronic medical record; DHMT, district health medical team.

### Ethical considerations

Ethical clearance to conduct this study was obtained from the Ministry of Health, National Health Research & Ethics Committee (No. ID65-2022).

## Results

### Flow of the search process and study characteristics

A total of 291 studies were searched from Google Scholar and PubMed, and 8 MOH and NAC documents were searched using Google. Articles were then screened and retrieved using the following combination of words: ‘community health information systems in Lesotho’ and ‘availability of health information systems in LMIC’ (*n* = 10). Eight articles and seven documents were found to be eligible and reviewed. Four documents were retrieved and used from MOH.

### Current availability of the community health information systems in Lesotho

**FIGURE 2:**
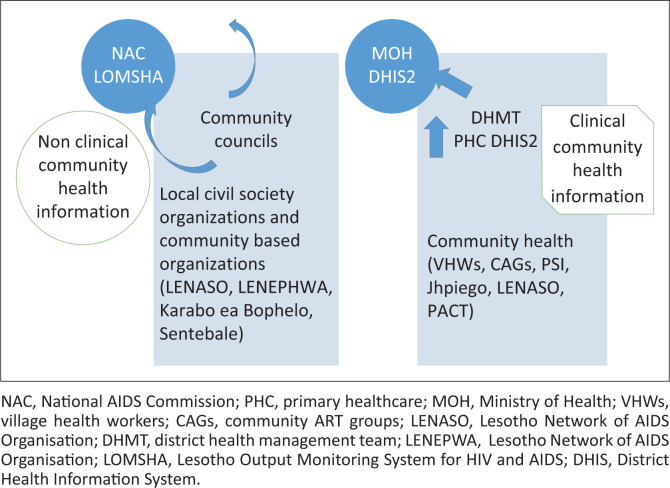
Available community health information system.

Figure 2 depicts that CSOs and community councils report non-clinical information to the NAC through LOMSHA. Village health workers, CAGs, and NGOs report clinical data to MOH through DHIS2. LOMSHA collects non-clinical information, and MOH community information system tools are being developed. As seen in Figure 2, there is no information-sharing link between LOMSHA and DHIS2.

#### Source of the community health information system in Lesotho

**FIGURE 3:**
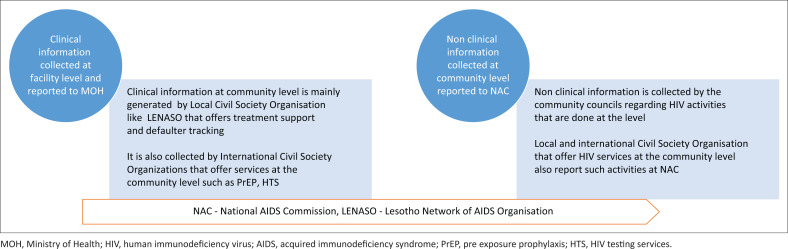
Source of clinical information as reported to the Ministry of Health and non-clinical as reported to National AIDS Commission.

Figure 3 shows sources of community health information comprising clinical and non-clinical information as reported to MOH and NAC. Community clinical information comes from local and international CSOs and is reported by health facilities through DHIS2 to the MOH. On the other hand, non-clinical information is gathered by CSOs and community councils, reported to the NAC and is contained in LOMSHA. Figure 3 shows no linkage between clinical and non-clinical information.

Table 2 illustrates two lists of information included in community clinical and non-clinical information.

**TABLE 2:** Community clinical and non-clinical information components.

Clinical information	Non-clinical information
HIV testing services	Tracking and linkages
Viral load suppression	Community education
Pre-exposure prophylaxis	Treatment support
Medicine pick-up	Home-based care
Adherence to medication	Psychosocial support
Lost to follow-up	Nutritional support
Mortality	Condom promotion and distribution
	Stigma and discrimination reduction
	Social and behavioural change

HIV, human immunodeficiency virus.

#### Clinical health information system at the community level in Lesotho

**FIGURE 4:**
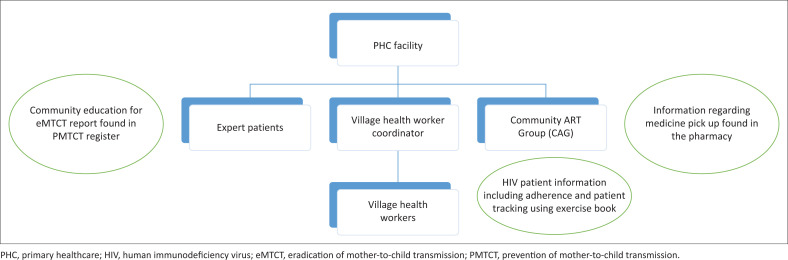
Clinical health information system at the community level.

Figure 4 illustrates the existing clinical health information system. In the existing system, there are no links between VHW, Gags, and expert patients. This indicates that the information about medicine pick-up and eradication prevention of mother to child information cannot be reported by VHW through coordinators, even though they report on adherence. Community clinical information is consolidated and reported by VHW coordinators which may lead to conflicting information reported.

#### Non-clinical health information system at the community level in Lesotho

**FIGURE 5:**
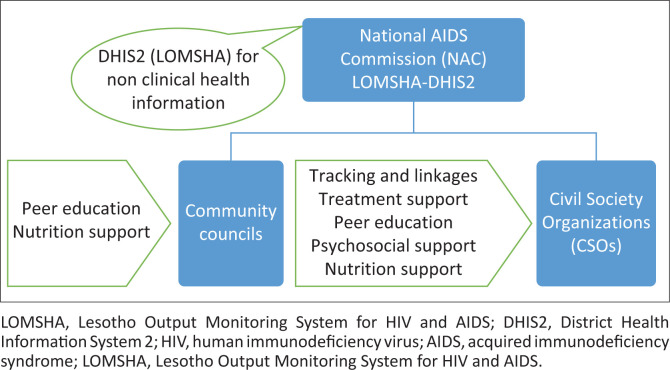
National AIDS Commission community structure.

Figure 5 displays non-clinical information collected at the community level by community councils and CSOs and it is then reported to NAC. It has the following components: peer education, nutritional support, treatment support, patient tracking, and linkages.

## Discussion

A CHIS exists in Lesotho. Clinical information from the community is reported to the PHC facility by VHWs, CAGs, expert patients, and local and international CSOs. Clinical information collected from the community by local and international CSOs is reported to MOH through DHIS2. The reports from VHWs and CAGs are not consolidated, may be contradictory, and are not included in the DHIS2. On the other hand, non-clinical community information is reported to NAC by community councils and CSOs. However, no linkages between DHIS2 and LOMSHA indicate that information is not shared between MOH and NAC.

As indicated, clinical information collected from the community by local and international CSOs is reported to MOH through DHIS2, but not information from VHWs and CAGs. The study of Tsoeu has established that DHIS2 is not effectively used.^26^ The study highlighted that reports from VHWs and CAGs are not consolidated as there are no links between them, and they may be contradictory. One study from Ethiopia indicated that community stakeholders and governments in LMICs lack community-based reporting mechanisms that provide the desired information.^1^ This study indicates that clinical information from the community reported by VHWs has no formal reporting tools. The study conducted in Lesotho indicated that in four districts of Lesotho, there are forms to fill out for M&E, HIV, and TB reporting.^28^ Some studies have found that CHWs are uniquely positioned to provide culturally relevant health-related information to HIS.^3,29^ In India, health information from the village level is included in the DHIS2.

On the other hand, non-clinical community information is reported to NAC by community councils and CSOs. However, there are no linkages between DHIS2 and LOMSHA. This is similar to the findings of Pepela and Otieno, which indicate that reported impediments to CHIS data utilisation include data quality, timeliness, and access to minimum data sets.^24^ Some studies noted that to aid decision-making and prevent unnecessary burdening of data providers, it is essential to identify and prioritise feasible and balanced measures across stakeholder needs, focusing on the minimal set necessary.^6,30^ In their study, King et al. added that historically, data-sharing projects have been ad hoc, inconsistent and limited in scope.^6^ However, the community health record framework for achieving collaboration and information exchange between health care, public health, and community organisations is iterative and participatory.^6^ Furthermore, this framework identifies concepts necessary for each aim and proposes an infrastructure to facilitate community health record development.^6^

The study implies that community-based health information is available and needs to be included in the existing health information systems to improve community-based decision-making regarding HIV and TB treatment outcomes. This has positive results for clinical and non-clinical CHIS improvements. Regarding health services, the findings imply that if clinical community health information from VHWs and CAGs is included in the PHC routine report, relevant interventions can be planned and executed locally. As for policy, Lesotho needs to prioritise the inclusion of clinical and non-clinical information, as massive amounts of information are collected at the community level, and if available to decision-makers at all levels, some of the interventions taken at the national level can be implemented at the facility level. Regarding future research, qualitative research should be conducted to reveal the real barriers that delay the inclusion of community health information into the existing health information systems.

## Conclusion

It can be concluded that CHISs are available in Lesotho in the form of DHIS2 from MOH and LOMSHA from NAC. However, clinical information from the community reported VHWs and CAGs are not reported on DHIS2, and there are no formal reporting tools. Again, there is no information sharing between MOH and NAC.

### Recommendations

Based on the findings, the following recommendations are made:

- MOH has to work on formalising reporting tools for VHWs and CAGs and creating formal linkages between community health players to avoid conflicting information.
- MOH and NAC have to formalise health information sharing to benefit both and to improve programming in these entities.

## References

[1] Mekonnen ZA, Chanyalew MA, Tilahun B, Gullslett MK, Mengiste SA. Lessons and implementation challenges of community health information system in LMICs: A scoping review of literature. Online J Public Health Inform. 2022;14(1):e5. 10.5210/ojphi.v14i1.12731 36457350 PMC9699826

[2] Center for Disease Control and Prevention. Health information and public health [homepage on the Internet]. Center for Disease Control and Prevention. 2018 [cited 2023 July 10]. Available from: https://www.cdc.gov/phlp/publications/topic/healthinformation.html.

[3] Cherrington A, Ayala GX, Elder JP, Arredondo EM, Fouad M, Scarinci I. Recognizing the diverse roles of community health workers in the elimination of health disparities: From paid staff to volunteers. NIH Public Access. 2010;20(2):189–194. PMC3695477 20503902

[4] Chewicha K, Azim T. Community health information system for family-centered health care: Scale-up in Southern Nations, Nationalities and People’s Region. Q Heal Bull. 2013;5(1):49–53.

[5] Debay M, Tantri A. On the design of community-based health information systems 2003. Meryland: USAID;2003.

[6] King RJ, Garrett N, Kriseman J, et al. A community health record: Improving health through multisector collaboration, information sharing, and technology. Prev Chronic Dis. 2016;13(9):1–9. 10.5888/pcd13.160101 PMC5027852 27609300

[7] Landsbergen D, Wolken G. Realizing the promise: Government information systems and the fourth generation of information technology. Public Adm Rev. 2001;61(2):206–220. 10.1111/0033-3352.00023

[8] Kingdom of Lesotho. National HIV and AIDS strategic plan 2018/19 – 2022/23 [homepage on the Internet]. 2018 [cited 2023 Dec 2]. Available from: https://www.prepwatch.org/resources/national-hiv-aids-strategic-plan-2018-19-2022-23/.

[9] Lesotho Ministry of Health. The village health program policy [homepage on the Internet]. 2020 [cited 2023 Dec 2]. Available from: https://extranet.who.int/countryplanningcycles/sites/default/files/public_file_rep/LSO_Lesotho_National-Health-Policy_2017.

[10] Jeremie N, Kaseje D, Olayo R, Akinyi C. Utilization of community-based health information systems in decision making and health action in Nyalenda, Kisumu County, Kenya. Univers J Med Sci. 2014;2(4):37–42. 10.13189/ujmsj.2014.020401

[11] Braun R, Catalani C, Wimbush J, Israelski D. Community health workers and mobile technology: A systematic review of the literature. PLoS One. 2013;8(6):4–9. 10.1371/journal.pone.0065772 PMC3680423 23776544

[12] USAID. Report of a technical consultation on information systems for community based HIV programs. Measure of Evaluation; 2009.

[13] National AIDS Commission (NAC). Lesotho Output Monitoring System for HIV And AIDS (LOMSHA) operation manual. 2017 [cited 2023 Dec 3]. Available from: https://www.nac.org.ls/coordination.

[14] Ha YP, Tesfalul MA, Littman-Quinn R, et al. Evaluation of a mobile health approach to tuberculosis contact tracing in Botswana. J Health Commun. 2016;21(10):1115–1121. 10.1080/10810730.2016.1222035 27668973 PMC6238947

[15] Otu A, Ebenso B, Okuzu O, Osifo-dawodu E. Using a mHealth tutorial application to change knowledge and attitude of frontline health workers to Ebola virus disease in Nigeria: A before-and-after study. Hum Resour Health. 2016;14(5):1–9. 10.1186/s12960-016-0100-4 26872824 PMC4751664

[16] Finocchario-Kessler S, Gautney BJ, et al. If you text them, they will come: Using the HIV infant tracking system to improve early infant diagnosis quality and retention in Kenya. NIH Public Access. 2014;28(3):1–16. 10.1097/QAD.0000000000000332 PMC4226133 24991904

[17] Ambia J, Mandala J. A systematic review of interventions to improve prevention of mother-to-child HIV transmission service delivery and promote retention. J Int AIDS Soc. 2016;19(1):1–11. 10.7448/IAS.19.1.20309 PMC4824870 27056361

[18] Mushamiri I, Luo C, Iiams-hauser C, Amor YB. Evaluation of the impact of a mobile health system on adherence to antenatal and postnatal care and prevention of mother-to-child transmission of HIV programs in Kenya. BMC Public Health. 2015;15(102):1–16. 10.1186/s12889-015-1358-5 25886279 PMC4328364

[19] Lwin MO, Vijaykumar S, Rathnayake VS, et al. A social media mHealth solution to address the needs of dengue prevention and management in Sri Lanka. J Med Internet Res. 2016;18(7):1–15. 10.2196/jmir.4657 PMC4947191 27369296

[20] Hampshire K, Mwase-Vuma T, Alemu K, et al. Informal mhealth at scale in Africa: Opportunities and challenges. World Dev. 2021;140:105257. 10.1016/j.worlddev.2020.105257 33814676 PMC7903241

[21] Birru E, Ndayizigiye M, McBain R, et al. Effects of primary healthcare reform on routine health information systems (RHISs): A mixed-methods study in Lesotho. BMJ Open. 2023;13(5):e071414. 10.1136/bmjopen-2022-071414 PMC10201246 37208141

[22] Mwase T, Eddie K, Julie D, Nomaphuthi H-K, Paul K-M, Taylor W. Lesotho health systems assessment. Bethesda, MD: Health Systems; 2010.

[23] Betjeman TJ, Soghoian SE, Foran MP. mHealth in sub-Saharan Africa. International Journal of Telemedicine and Applications. 2013;2013:482324. 10.1155/2013/482324 24369460 PMC3867872

[24] Ngcobo S, Scheepers S, Mbatha N, Grobler E, Rossouw T. (2022). Roles, barriers, and recommendations for community health workers providing community-based HIV Care in Sub-Saharan Africa: A review. AIDS Patient Care and STDs. 2022;36(4):130–144. 10.1089/apc.2022.0020 35438523 PMC9057893

[25] Källander K, Tibenderana JK, Akpogheneta OJ. Mobile health (mHealth) approaches and lessons for increased performance and retention of community health workers in low-and middle-income countries: A review. Journal of Medical Internet Research. 2013;15(1):e17. 10.2196/jmir.2130 23353680 PMC3636306

[26] Tsoeu S. Utilization of district health information system 2 by healthcare workers in Lesotho. Vol. 33, Braz Dent J. 2022.

[27] National AIDS Commission (NAC). Lesotho HIV and AIDS M&E Plan (2018–2023) [homepage on the Internet]. 2018 [cited 2023 Nov 3]. Available from: https://www.prepwatch.org/resources/national-hiv-aids-strategic-plan-2018-19-2022-23/.

[28] Ngilangwa DP, Mgomella GS. Factors associated with retention of community health workers in maternal, newborn and child health programme in Simiyu Region, Tanzania. African J Prim Heal Care Fam Med. 2018;10(1):1–8. 10.4102/phcfm.v10i1.1506 PMC6131709 30198284

[29] Pepela WD, Otieno GWO. Community health information system utility: A case of Bungoma County Kenya. Int Res J Public Environ Heal. 2016;3(4):75–86. 10.15739/irjpeh.16.010

[30] Garg R, Garg A. District health information system (DHIS2) software in India. Adv Comput Sci Inf Technol. 2015;2(10):39–42.

